# Development of Obsessive-Compulsive Symptoms Following Abrupt Discontinuation of Venlafaxine

**DOI:** 10.3389/fpsyt.2020.00032

**Published:** 2020-02-12

**Authors:** Maximilian Gahr, Christoph Hiemke, Markus Alexander Kölle

**Affiliations:** ^1^Department of Psychiatry and Psychotherapy III, University of Ulm, Ulm, Germany; ^2^Department of Psychiatry and Psychotherapy, University Medical Center of Mainz, Mainz, Germany

**Keywords:** depression, serotonin, selective serotonin reuptake inhibitors, suicidality, tapering

## Abstract

Withdrawal symptoms after discontinuation of antidepressants are common and have long been known. Typical symptoms after dose reduction or discontinuation of antidepressants are dizziness, drowsiness, headache, flu-like symptoms, hyperarousal, imbalance, insomnia, irritability, and nausea. Rebound, relapse, or recurrence associated with the underlying mental disorder may also occur. The occurrence of obsessive-compulsive symptoms (OCS) following abrupt discontinuation of antidepressants have not yet been reported. Here we report the development of OCS (obsessional suicidal thoughts) in a patient with major depressive disorder and absence of a previous obsessive-compulsive disorder following abrupt discontinuation of venlafaxine. Treatment with escitalopram facilitated remission of OCS. We discuss a possible causal link between abrupt discontinuation of venlafaxine and development of OCS under consideration of pathophysiologic aspects regarding obsessive compulsive disorders, the chronological sequence of symptoms in the present case, and pharmacodynamic and -kinetic aspects. Our case report suggests the possibility of the occurrence of obsessive-compulsive symptoms following abrupt discontinuation of venlafaxine.

## Introduction and Background

Antidepressant withdrawal phenomena have long been known (1). The antidepressant discontinuation syndrome, not only regarding selective serotonin reuptake inhibitors (SSRI), was defined (2, 3) which is also listed in the Diagnostic and Statistical Manual of Mental Disorders, Fifth Edition (DSM-5) (4). However, withdrawal after discontinuation of antidepressants or antidepressant withdrawal is the currently used term (5, 6) and a new classification of SSRI withdrawal was created (7). In this regard it is important to take into account that the consideration and evaluation of symptoms following discontinuation of antidepressants has switched from “antidepressant discontinuation syndrome” to “antidepressant withdrawal syndrome” (5, 6). There are symptoms that occur rather immediate after discontinuation of the agent as dizziness, drowsiness, headache, flu-like symptoms, hyperarousal, imbalance, insomnia, irritability, or nausea (5) and delayed events that may occur later as rebound, relapse, or recurrence associated with the underlying mental disorder (8). In addition, the importance of the distinction between new withdrawal symptoms, rebound, and persistent post-withdrawal disorders was emphasized (7). Based on results of a current systematic review withdrawal incidence rates range from 27 to 86% with a weighted average of 56% (9). Slow tapering may also result in withdrawal (5, 6). Discontinuation of selective serotonin-noradrenaline reuptake inhibitors as venlafaxine (and desvenlafaxine) or duloxetine may also cause withdrawal symptoms (6). There is even evidence that venlafaxine is associated with a higher risk for the occurrence of withdrawal symptoms (10), both in comparison with duloxetine (11) and SSRI (escitalopram, sertraline) (12–14). In addition, severe forms of withdrawal phenomena appear to occur more frequently in association with venlafaxine, and abrupt discontinuation of venlafaxine is associated with a higher incidence for withdrawal symptoms than tapering (13). In principle, withdrawal symptoms related to venlafaxine/desvenlafaxine resemble those of SSRI (7, 10, 13, 15). Here we report on a patient with major depressive disorder who developed obsessive-compulsive symptoms following abrupt discontinuation of venlafaxine.

## Case Presentation

The 25-year-old male patient was admitted to our psychiatric day hospital due to depressive symptoms comprising anhedonia, depressed mood, loss of energy, sleep disturbances (troubles falling asleep and staying asleep), subjective cognitive impairment (impaired attention and indecisiveness), and drop in self-esteem, present for about 2 months. Suicidal tendencies were not present. The patient featured moderate functional impairment and had not been able to work for 1 month (merchant in wholesale and foreign trade). Three years earlier the patient had developed a first depressive episode that had been treated successfully with sertraline (100 mg per day) in combination with outpatient psychotherapy (cognitive-behavioral therapy). After remission of the first depressive episode the patient was asymptomatic under monotherapy with sertraline (100 mg per day) until 2 months prior to the current admission. Taking into account the absence of a current or past use of any psychotropic agent/illicit drug, absence of any physical illness (body mass index 25,2 kg/m^2^), inconspicuous physical examination, laboratory tests (including drug screening in urine), and cranial magnetic resonance tomography a major depressive disorder, recurrent episode with currently moderate severity was diagnosed according to DSM-5 criteria (DSM-5 296.32) (4); no specific rating scales or interviews were used for diagnosis; the diagnosis was formulated by an experienced psychiatrist and consultant (MG) based on personal clinical examination and interview. The patient received a multiprofessional treatment including cognitive behavioral therapy, regular psychiatric examinations, support of social workers, and additional therapies (e.g. sport, art, and music therapy). At the time of admission the antidepressive psychopharmacotherapy was switched to venlafaxine. Sertraline was slowly reduced and venlafaxine (extended release) was administered simultaneously starting with 37,5 mg per day; the dose of venlafaxine was gradually increased. The cross-tapering phase took 10 days and was well tolerated. After discontinuation of sertraline, venlafaxine was gradually increased up to a daily dose of 150 mg. No adverse drug reactions (ADR) or withdrawal phenomena occurred. After 3 weeks of monotherapy with venlafaxine 150 mg per day (approximately 5 weeks after admission to the day hospital) the patient reported significant improvement in depressive symptoms. However, he complained marked restlessness and hyperhidrosis that had gradually developed during the past 2 weeks (approximately 1 week after monotherapy with venlafaxine in a daily dose of 150 mg). The patient stated to suffer heavily from these ADR. Thereupon he refused continuation of venlafaxine due to restlessness and hyperhidrosis and requested abrupt cessation. Although he was sufficiently informed about the potential occurrence of withdrawal symptoms he insisted on immediate cessation of venlafaxine. Hence, venlafaxine was stopped and a temporary *pro re nata* (PRN) medication with lorazepam (0.5 mg) was established which the patient also refused. Approximately 2 days after cessation of venlafaxine the patient developed headache, mild nausea, dizziness, and an increase of restlessness and hyperhidrosis; these symptoms receded approximately 8 days after discontinuation of venlafaxine. Four days after discontinuation of venlafaxine the patient additionally reported the development of increasing intrusive, recurrent, and unwanted suicidal thoughts that were present nearly the whole day. The patient clearly stated that he did not want to harm himself or commit suicide, however was afraid to do so due to the intensity and intrusiveness of the mentioned thoughts. He stated that these thoughts would make no sense to him as he was actually happy that his depression had markedly improved. The patient reported that he had repeatedly tried to resist these thoughts (e.g. stopping to think them), which had caused even an increase in fear of harming himself. He appeared significantly impaired and frightened by this newly developed phenomenon and was not able to suppress those thoughts. Symptoms of depression did not worsen after discontinuation of venlafaxine and onset of obsessive-compulsive symptoms. As true suicidal tendencies were not found the mentioned thoughts were classified as compulsions/obsessional thoughts and evaluated as a possible consequence of the abrupt cessation of venlafaxine. According to the anamnestic information by the patient this was the first episode of obsessive-compulsive symptoms and the patient's family anamnesis was negative regarding this disorder. The patient was informed about the evaluation and SSRI treatment was recommended. Escitalopram 10 mg per day was started 9 days after discontinuation of venlafaxine. After 7 days of treatment with escitalopram compulsions were regressive and subsided completely following 12 days of monotherapy with escitalopram 10 mg per day. The improvement of depressive symptoms that had developed under venlafaxine sustained until the end of the treatment in the psychiatric day hospital under monotherapy with escitalopram. Concerning ADRs restlessness was not present under escitalopram. A mild degree of hyperhidrosis, yet not as pronounced as under venlafaxine, had developed. The patient was discharged 2 weeks after initialization of escitalopram. Psychotherapy for maintenance of remission and relapse prevention was recommended, however refused by the patient. A further follow 9 months after discharge demonstrated a stable remission under monotherapy with escitalopram 10 mg per day without any depressive or obsessional symptoms.

## Discussion

Type and course of the symptoms which the patient had developed 2 days after abrupt discontinuation of venlafaxine are characteristic of venlafaxine withdrawal (6, 10, 13, 15). However, development of obsessive-compulsive symptoms after discontinuation of venlafaxine has not yet been reported (6). Rebound, relapse, or recurrence of symptoms associated with the underlying mental disorder may also occur following discontinuation of antidepressants (1, 2, 6). In the present case, however, an obsessive-compulsive disorder was neither present in the past nor reason for the current treatment with venlafaxine; thus, the reported development of compulsions/obsessional thoughts should not be interpreted as a relapse- or rebound-related phenomenon in the context of the underlying mental disorder, particularly since the patient featured obsessive-compulsive symptoms neither on time of admission nor in the past. Indeed, suicidal tendencies are typically associated with depression and have also been reported as symptoms following discontinuation of venlafaxine (6, 16). In the published cases, however, “true” suicidal ideation was present. By contrast, from a psychopathological point of view, the phenomena reported by the patient in the present case (suicidal thoughts, unwanted and intrusive thoughts to harm himself or to commit suicide) should not be classified as “true” suicidality, that is indeed typical of major depressive disorder, however usually presents with the factual intention to self-harm or suicide (“ego syntonic phenomenon”); in the present case the patient described his suicidal thoughts as intrusive, repetitive, and—most important—unwanted and clearly stated that he did not want to commit suicide or to harm himself; moreover, he stated that these thoughts would make no sense to him. In this regard, the patient's suicidal thoughts featured characteristics of obsessional symptoms. To summarize, according to the criteria presented by Chouinard and Chouniard our patient may have presented new withdrawal symptoms related to discontinuation of venlafaxine (7).

A causal relationship between the abrupt discontinuation of venlafaxine and the occurrence of compulsions cannot be proven. Yet, the chronological sequence of both events, remission of compulsions after initiation of treatment with escitalopram, and pharmacodynamic and -kinetic considerations speak in favor of a possible causal link. Among several other etiologically relevant factors there is evidence for a dysfunction of the serotonergic system in obsessive-compulsive disorder (serotonin hypothesis) (17) and serotonin reuptake inhibitors are used successfully in the treatment of this disorder (18). Both, venlafaxine and escitalopram feature moderate respectively high affinity to the serotonin transporter (5-HTT) and potent serotonin reuptake blocking properties (19, 20). Abrupt discontinuation of venlafaxine that features a comparatively short elimination half-life [venlafaxine extended release: venlafaxine 14–18 h, O-desmethylvenlafaxine 10–17 h (21)] may have altered the central serotonergic neurotransmission, presumably also in the orbitofrontal cortex that is supposed to be a key structure in the hypothesized serotonin-based pathophysiology of obsessive-compulsive disorders (17). Treatment with escitalopram may have rebalanced serotonergic neurotransmission and remission of compulsions following 12 days of treatment with escitalopram is in line with the long elimination half-life of escitalopram of 27–33 h (22). However, neither the pathophysiology of obsessive-compulsive disorders nor the neurobiological mechanisms related to discontinuation of antidepressants/antidepressant withdrawal are currently sufficiently understood; neuronal processes etiologically involved in obsessive-compulsive disorders may not be relevant in drug-related obsessive-compulsive symptoms, particularly antidepressant withdrawal. Moreover, the serotonergic hypothesis regarding the mechanism of action of antidepressants was questioned (23). Therefore, a further discussion of potential pathophysiologic mechanisms related to the reported drug-related phenomenon is too speculative, and further research is necessary in this field.

The present case suggests the possibility of the occurrence of obsessive-compulsive symptoms following abrupt discontinuation of venlafaxine. Furthermore, it emphasizes further aspects of behavioral toxicity related to antidepressive psychopharmacotherapy (24).

## Ethics Statement

Written informed consent was obtained from the patient for publication of this case report.

## Author Contributions

MG treated the patient, wrote the manuscript, performed the literature search and evaluated the clinical course of the patient against the background of the relevant literature. MK treated the patient, corrected the manuscript and wrote the discussion. CH wrote and corrected the manuscript and discussed the clinical findings under due consideration of the relevant literature.

## Conflict of Interest

The authors declare that the research was conducted in the absence of any commercial or financial relationships that could be construed as a potential conflict of interest.
